# Supplementary material for: Cannabinoid use generalizes stress responses: involvement of astrocyte plasticity and activation of matrix metalloproteinases in the nucleus accumbens core
Source: Mol Psychiatry. 2026 Jun 11;31(10):5979–92. doi: 10.1038/s41380-026-03687-0 (PMC13569448; doi:10.1038/s41380-026-03687-0)
Supplement: Supplementary file 1 [file 41380_2026_3687_MOESM1_ESM.docx]

|  | Veh | | | | | | THC+CBD | | | | | |
| --- | --- | --- | --- | --- | --- | --- | --- | --- | --- | --- | --- | --- |
|  | Sham-CS | | Stress-NS | | Stress-CS | | Sham-CS | | Stress-NS | | Stress-CS | |
|  | K2 | P value | K2 | P value | K2 | P value | K2 | P value | K2 | P value | K2 | P value |
| Astro-Syn (%ROI) | 14.1 | 0.0009 | 22.52 | <0.0001 | 6.38 | 0.04 | 10.12 | 0.006 | 24.88 | <0.0001 | 5.86 | 0.053 |
| Synapsin-I | 4.1 | 0.128 | 2.07 | 0.35 | 4.97 | 0.08 | 6.224 | 0.044 | 29.65 | <0.0001 | 1.359 | 0.5 |
| Surface Area | 4.16 | 0.125 | 9.83 | 0.007 | 15.43 | 0.0004 | 16.92 | 0.0002 | 4.94 | 0.084 | 8.3 | 0.016 |
| Volume | 18.3 | 0.0001 | 13.68 | 0.0011 | 44.12 | <0.0001 | 9.59 | 0.008 | 18.16 | 0.0001 | 8.1 | 0.0173 |

**Table S1. Data distribution.** Data were analyzed by Linear Mixed Model test when populations were found not to be normally distributed using D’Agostino-Pearson normality test.
