# Supplementary material for: Cannabinoid use generalizes stress responses: involvement of astrocyte plasticity and activation of matrix metalloproteinases in the nucleus accumbens core
Source: Mol Psychiatry. 2026 Jun 11;31(10):5979–92. doi: 10.1038/s41380-026-03687-0 (PMC13569448; doi:10.1038/s41380-026-03687-0)
Supplement: Supplementary file 2 [file 41380_2026_3687_MOESM2_ESM.docx]

| **Figure 1: Acute restraint stress potentiated THC+CBD use in males** | | | | |
| --- | --- | --- | --- | --- |
|  | Total n | N break down | Statistical analysis | |
| B | 23 | Sham=8, Stress=15 | **Two-way repeated measures ANOVA**  *Stress: F_1,21_=4.547 p=0.0449, ƞ^2^=0.08  *Days: F_9,189_=3.001, p=0.0023, ƞ^2^=0.058  Stress x Days: F_9,189_=1.094 p=0.3688, ƞ^2^=0.02 | |
| C |  |  | **Two-way repeated measures ANOVA**  *Stress: F_1,21_=5.404 p=0.0302, ƞ^2^=0.1  Days: F_9,189_=2.198, p=0.0239, ƞ^2^=0.04  Stress x Days: F_9,189_=1.763 p=0.0777, ƞ^2^=0.03 | |
| D |  |  | **Two-way repeated measures ANOVA**  Stress: F_1,21_=2.933 p=0.1015, ƞ^2^=0.06  Days: F_2.992,68.83_=0.1866, p=0.9047, ƞ^2^=0.004  Stress x Days: F_4,84_=0.189 p=0.1197, ƞ^2^=0.04 | |
| E | 22 | Sham=6, Stress=16 | **Two-way repeated measures ANOVA**  Stress: F_1,20_=0.3335 p=0.5701, ƞ^2^=0.006  *Days: F_2.746,54.93_=8.233, p=0.0002, ƞ^2^=0.17  Stress x Days: F_9,180_=1.491 p=0.1542, ƞ^2^=0.03 | |
| F |  |  | **Two-way repeated measures ANOVA**  Stress: F_1,20_=0.1854 p=0.1884, ƞ^2^=0.026  *Days: F_3.377,67.53_=10.41, p<0.0001, ƞ^2^=0.23  Stress x Days: F_9,180_=1.714 p=0.166, ƞ^2^=0.038 | |
| G |  |  | **Two-way repeated measures ANOVA**  Stress: F_1,20_=0.4475 p=0.5112, ƞ^2^=0.01  Days: F_3.123,62.47_=0.5352, p=0.667, ƞ^2^=0.01  Stress x Days: F_4,80_=1.057 p=0.3835, ƞ^2^=0.02 | |
| **Figure 2: THC+CBD generalized stress responses in males** | | | | |
|  | Total n | N break down | Statistical analysis | |
| A | 45 | Veh  Sham-CS =6; Stress-NS =7; Stress-CS =9  THC+CBD  Sham-CS =8; Stress-NS =7; Stress-CS =8 | **Two-way ANOVA**  Drug: F_1,39_=2.083, p=0.1569 Stress: F_2,39_=3.108, p=0.0559  *Drug x Stress: F_2,39_=9.8, p<0.0001 | |
| B |  |  | **Two-way ANOVA**  *Drug: F_1,39_=41.35, p<0.0001 Stress: F_2,39_=52.4, p=0.5962 Drug x Stress: F_2,39_=68.34, p=0.5108 | |
| C |  |  | **Two-way ANOVA**  *Drug: F_1,39_=4.842, p=0.0338 Stress: F_2,39_=11.4, p=0.8926 Drug x Stress: F_2,39_=1.241, p=0.3002 | |
| D |  |  | **Two-way ANOVA**  Drug: F_1,39_=0.0007956, p=0.9776 *Stress: F_2,39_=9.835, p=0.0003 Drug x Stress: F_2,39_=2.382, p=0.1056 | |
| E |  |  | **Multiple Chi-square; Corrected p =0.0071**  *Sham-CS veh vs Stress-CS veh: X^2^_(3)_ =34.06, p<0.0001  Sham-CS veh vs Stress-NS veh: X^2^_(3)_ =5.64, p=0.1305  *Sham-CS THC vs Stress-CS THC: X^2^_(3)_ =34.06, p<0.0001  *Sham-CS THC vs Stress-NS THC: X^2^_(3)_ =113.2, p<0.0001  *Sham-CS veh vs Sham-CS THC: X^2^_(3)_ =974.4, p<0.0001  *Stress-NS veh vs Stress-NS THC: X^2^_(3)_ =70.45, p<0.0001  *Stress-CS veh vs Stress-CS THC: X^2^_(3)_ =106.5, p<0.0001 | |
| **Figure 3: THC+CBD use reduced the astroglial coverage of synapses in NAcore but potentiated the synaptic insulation by astrocytes after the stress-CS exposure in males** | | | | |
|  | Total n | N break down | Statistical analysis | |
| B | 31 | Veh  Sham-CS =4; Stress-NS =5; Stress-CS =5  THC+CBD  Sham-CS =4; Stress-NS =7; Stress-CS =6 | **LMM**  Number of Value: 268  Random effects  σ^2^animal=0.179; σ^2^residual=0.617; ICC= 0.22  Fixed effects: Treatment group  * X^2^_(5)_= 50.37, p<0.0001, R^2^_m_=0.3351 | |
| C |  |  | **Right: Multiple Chi-square; Corrected p =0.0071**  *Sham-CS veh vs Stress-CS veh: X^2^_(2)_ =16, p=0.0003  *Sham-CS veh vs Stress-NS veh: X^2^_(2)_ =41.94, p<0.0001  *Sham-CS THC vs Stress-CS THC: X^2^_(2)_ =36.02, p<0.0001  *Sham-CS THC vs Stress-NS THC: X^2^_(2)_ =11.8, p=0.0027  *Sham-CS veh vs Sham-CS THC: X^2^_(2)_ =32.33, p<0.0001  Stress-NS veh vs Stress-NS THC: X^2^_(2)_ =6.437, p=0.04  *Stress-CS veh vs Stress-CS THC: X^2^_(2)_ =19.2, p<0.0001 | |
| D |  |  | Veh  *Spearman r=-0.476, p=0.0435 | THC+CBD  Spearman r=0.01982, p=0.4705 |
| E |  |  | Veh  Pearson r=0.4803, p=0.4352 | THC+CBD  *Pearson r=-0.444, p=0.0369 |
| F |  |  | **LMM**  Number of Value: 268  Random effects  σ^2^animal=0.041; σ^2^residual=0.0952; ICC= 0.04  Fixed effects: Treatment group  * X^2^_(5)_= 25.9573, p=0.0001, R^2^_m_=0.2369 | |
| G |  |  | **Right: Multiple Chi-square; Corrected p =0.0071**  Sham-CS veh vs Stress-CS veh: X^2^_(2)_ =3.24, p=0.1979  Sham-CS veh vs Stress-NS veh: X^2^_(2)_ =7.353, p=0.0253  *Sham-CS THC vs Stress-CS THC: X^2^_(2)_ =17.5, p=0.0002  *Sham-CS THC vs Stress-NS THC: X^2^_(2)_ =44.98, p<0.0001  Sham-CS veh vs Sham-CS THC: X^2^_(2)_ =9.881, p=0.0072  *Stress-NS veh vs Stress-NS THC: X^2^_(2)_ =13.1, p=0.0014  *Stress-CS veh vs Stress-CS THC: X^2^_(2)_ =21.82, p<0.0001 | |
| **Figure 4: THC+CBD use induced astrocyte atrophy after the stress-CS exposure in males** | | | | |
|  | Total n | N break down | Statistical analysis | |
| A | 31 |  | **LMM**  Number of Value: 268  Random effects  σ^2^animal=0.031; σ^2^residual=0.1613; ICC= 0.16  Fixed effects: Treatment group  * X^2^_(5)_= 26.15, p=0.0001, R^2^_m_=0.184 | |
| B |  | Veh  Sham-CS =4; Stress-NS =5; Stress-CS =5  THC+CBD  Sham-CS =4; Stress-NS =7; Stress-CS =6 | **Right: Multiple Chi-square; Corrected p =0.0071**  Sham-CS veh vs Stress-CS veh: X^2^_(2)_ =3.413, p=0.1815  Sham-CS veh vs Stress-NS veh: X^2^_(3)_ =7.338, p=0.0255  *Sham-CS THC vs Stress-CS THC: X^2^_(2)_ =15.19, p=0.0005  Sham-CS THC vs Stress-NS THC: X^2^_(2)_ =5.974, p=0.0504  *Sham-CS veh vs Sham-CS THC: X^2^_(2)_ =14.33, p=0.0008  Stress-NS veh vs Stress-NS THC: X^2^_(2)_ =3.671, p=0.1596  *Stress-CS veh vs Stress-CS THC: X^2^_(2)_ =44.01, p<0.0001 | |
| C |  |  | **LMM**  Number of Value: 268  Random effects  σ^2^animal=0.048; σ^2^residual=0.2427; ICC= 0.165  Fixed effects: Treatment group  * X^2^_(5)_= 32.86, p<0.0001, R^2^_m_=0.2228 | |
| D |  |  | **Multiple Chi-square; Corrected p =0.0071**  *Sham-CS veh vs Stress-CS veh: X^2^_(2)_ =9.965, p=0.0069  Sham-CS veh vs Stress-NS veh: X^2^_(3)_ =1.733, p=0.4204  *Sham-CS THC vs Stress-CS THC: X^2^_(2)_ =13.33, p=0.0013  Sham-CS THC vs Stress-NS THC: X^2^_(2)_ =7.347, p=0.0254  Sham-CS veh vs Sham-CS THC: X^2^_(2)_ =5.672, p=0.0587  Stress-NS veh vs Stress-NS THC: X^2^_(2)_ =4.546, p=0.1030  *Stress-CS veh vs Stress-CS THC: X^2^_(2)_ =64.4, p<0.0001 | |
| E |  |  | Veh  *Pearson r=0.486, p=0.039 | THC+CBD  *Pearson r=-0.512, p=0.0177 |
| F |  |  | Veh  Pearson r=0.06722, p=0.4146 | THC+CBD  *Pearson r=-0.612, p=0.0039 |
| **Figure 5: THC+CBD use potentiated the MMP-2,9 activity induced by the stress-CS in males** | | | | |
|  | Total n | N break down | Statistical analysis | |
| B | 37 | Veh  Sham-CS =6; Stress-NS =7; Stress-CS =7  THC+CBD  Sham-CS =6; Stress-NS =5; Stress-CS =6 | **Two-way ANOVA**  *Drug: F_1,31_=54.83, p<0.0001, ƞ^2^=0.33 *Stress: F_2,31_=33.03, p<0.0001, ƞ^2^=0.39  *Drug x Stress: F_2,31_=7.686, p=0.0019, ƞ^2^=0.09 | |
| C |  |  | Veh  *Pearson r=0.4848, p=0.0303 | THC+CBD  *Pearson r=-0.4557, p=0.033 |
| D |  |  | Veh  Spearman r=0.2816, p=0.1145 | THC+CBD  *Spearman r=0.5403, p=0.0135 |
| E | 25 | Veh  Sham-CS =4; Stress-NS =5; Stress-CS =4  THC+CBD  Sham-CS =3; Stress-NS =5; Stress-CS =4 | Veh  Pearson r=0.1214, p=0.3464 | THC+CBD  *Pearson r=0.7504, p=0.0049 |
| F |  |  | Veh  Pearson r=0.3617, p=0.1123 | THC+CBD  *Pearson r=-0.547, p=0.0328 |
| **Figure 6: MMP-2 regulated the multipartite synaptic plasticity following THC+CBD use** | | | | |
|  | Total n | N break down | Statistical analysis | |
| B | 20 | CS-DMSO =8; CS-9i =6; CS-2i =6 | **One-way ANOVA**  F_2,16_=1.183, p=0.3318 | |
| C |  |  | **LMM (left)**  Number of Value: 168  Random effects  σ^2^animal=0.161; σ^2^residual=0.3818; ICC= 0.29  Fixed effects: Treatment group  * X^2^_(2)_= 18.08, p<0.0001, R^2^_m_=0.2039  **Multiple Chi-square; Corrected p =0.017 (right)**  *CS-DMSO vs CS-9i: X^2^_(2)_ =18.13, p=0.0001  *CS-DMSO vs CS-2i: X^2^_(2)_ =47.9, p<0.0001  *CS-2i vs CS-9i: X^2^_(2)_ =10.51, p=0.0052 | |
| D |  |  | **LMM (left)**  Number of Value: 168  Random effects  σ^2^animal=0.016; σ^2^residual=0.1355; ICC= 0.10  Fixed effects: Treatment group  * X^2^_(2)_= 7.58, p=0.022, R^2^_m_=0.08  **Multiple Chi-square; Corrected p =0.017 (right)**  CS-DMSO vs CS-9i: X^2^_(2)_ =2.43, p=0.31  *CS-DMSO vs CS-2i: X^2^_(2)_ =24.72, p<0.0001  *CS-2i vs CS-9i: X^2^_(2)_ =17.88, p=0.0001 | |
| E |  | CS-DMSO =8; CS-9i =6; CS-2i =6 | **LMM (left)**  Number of Value: 168  Random effects  σ^2^animal=0.022; σ^2^residual=0.0946; ICC= 0.18  Fixed effects: Treatment group  *X^2^_(2)_= 5.4, p=0.067, R^2^_m_=0.06  **Multiple Chi-square; Corrected p =0.017 (right)**  CS-DMSO vs CS-9i: X^2^_(2)_ =2.52, p=0.2837  *CS-DMSO vs CS-2i: X^2^_(2)_ =24.75, p<0.0001  *CS-2i vs CS-9i: X^2^_(2)_ =27.28, p<0.0001 | |
| F |  |  | **LMM (left)**  Number of Value: 168  Random effects  σ^2^animal=0.045; σ^2^residual=0.01807; ICC= 0.19  Fixed effects: Treatment group  X^2^_(2)_= 5.8, p=0.054, R^2^_m_=0.1  **Multiple Chi-square; Corrected p =0.017 (right)**  CS-DMSO vs CS-9i: X^2^_(2)_ =1.74, p=0.42  *CS-DMSO vs CS-2i: X^2^_(2)_ =23.3, p<0.0001  *CS-2i vs CS-9i: X^2^_(2)_ =35.55, p<0.0001 | |
| G |  |  | **Multiple Chi-square; Corrected p =0.017**  *CS-DMSO vs CS-9i: X^2^_(2)_ =10.32, p=0.0058  *CS-DMSO vs CS-2i: X^2^_(2)_ =10.6, p=0.005  *CS-2i vs CS-9i: X^2^_(2)_ =96.79, p<0.0001 | |
| **Figure S1: Acute restraint stress increased the discrimination index in female THC+CBD rats** | | | | |
|  | Total n | N break down | Statistical analysis | |
| A | 17 | Sham=5, Stress=12 | **Two-way repeated measure ANOVA**  Stress: F_1,15_=0.2947, p=0.5952, ƞ^2^=0.07  *Days: F_2.897,43.45_=3.013, p=0.0417, ƞ^2^=0.09  Stress x Days: F_9,135_=0.9991, p=0.4439, ƞ^2^=0.03 | |
| B |  |  | **Two-way repeated measure ANOVA**  Stress: F_1,15_=0.1881 p=0.6706, ƞ^2^=0.004  *Days: F_9.135_=2.068, p=0.0366, ƞ^2^=0.07  Stress x Days: F_9,135_=0.7586, p=0.6547, ƞ^2^=0.02 | |
| C |  |  | **Two-way repeated measure ANOVA**  *Stress: F_1,15_=13.53, p=0.0022, ƞ^2^=0.26  Days: F_3.019,45.29_=0.7343, p=0.5378, ƞ^2^=0.02  Stress x Days: F_4,60_=0.2228 p=0.9247, ƞ^2^=0.06 | |
| D | 18 | Sham=5, Stress=13 | **Two-way repeated measure ANOVA**  Stress: F_1,16_=1.72, p=0.2082, ƞ^2^=0.01  *Days: F_3.024,48.39_=9.929, p<0.0001, ƞ^2^=0.28  Stress x Days: F_9,144_=0.2052 p=0.9932, ƞ^2^=0.0058 | |
| E |  |  | **Two-way repeated measure ANOVA**  Stress: F_1,10_=4.287 p=0.0652, ƞ^2^=0.05  *Days: F_3.793,37.93_=14.62, p<0.0001, ƞ^2^=0.43  Stress x Days: F_9,90_=1.843, p=0.0887, ƞ^2^=0.05 | |
| F |  |  | **Two-way repeated measure ANOVA**  Stress: F_1,16_=0.2876 p=0.5991, ƞ^2^=0.008  Days: F_3.465,55.44_=1.365, p=0.2609, ƞ^2^=0.03  Stress x Days: F_4,64_=0.7953, p=0.5326, ƞ^2^=0.02 | |
| **Figure S2: Coping strategies in females** | | | | |
|  | Total n | N break down | Statistical analysis | |
| A | 33 | Veh  Sham-CS =5; Stress-NS =5; Stress-CS =6  THC+CBD  Sham-CS =5; Stress-NS =6; Stress-CS =6 | **Two-way ANOVA**  Drug: F_1,27_=1.777, p=0.1937, ƞ^2^=0.05 Stress: F_2,27_=1.903, p=0.1687, ƞ^2^=0.11  Drug x Stress: F_2,27_=0.447, p=0.6456, ƞ^2^=0.02 | |
| B |  |  | **Two-way ANOVA**  Drug: F_1,27_=0.0049, p=0.9444, ƞ^2^=0.0001 Stress: F_2,27_=0.1383, p=0.8714, ƞ^2^=0.0009  Drug x Stress: F_2,27_=0.2064, p=0.8148, ƞ^2^=0.001 | |
| C |  |  | **Two-way ANOVA**  Drug: F_1,27_=2.744, p=0.1092, ƞ^2^=0.08 Stress: F_2,27_=0.8464, p=0.44, ƞ^2^=0.05  Drug x Stress: F_2,27_=0.6617, p=0.5242, ƞ^2^=0.04 | |
| D |  |  | **Two-way ANOVA**  Drug: F_1,27_=0.2721, p=0.6062, ƞ^2^=0.009 Stress: F_2,27_=0.3789, p=0.6882, ƞ^2^=0.02  Drug x Stress: F_2,27_=0.2311, p=0.7952, ƞ^2^=0.01 | |
| **Figure S3: THC+CBD use potentiated the synaptic insulation by astrocytes after the stress-CS exposure in females** | | | | |
|  | Total n | N break down | Statistical analysis | |
| A | 23 | Veh  Sham-CS =4; Stress-NS =4; Stress-CS =3  THC+CBD  Sham-CS =4; Stress-NS =5; Stress-CS =3 | **LMM (left)**  Number of Value: 175  Random effects  σ^2^animal=0.123; σ^2^residual=0.6116; ICC= 0.17  Fixed effects: Treatment group  *X^2^_(5)_= 30.3119, p<0.0001, R^2^_m_=0.2715 | |
| B |  |  | **Multiple Chi-square; Corrected p =0.0071**  *Sham-CS veh vs Stress-CS veh: X^2^_(2)_ =50.04, p<0.0001  Sham-CS veh vs Stress-NS veh: X^2^_(3)_ =9.801, p=0.00074  *Sham-CS THC vs Stress-CS THC: X^2^_(2)_ =48.65, p<0.0001  Sham-CS THC vs Stress-NS THC: X^2^_(2)_ =0.4283 p=0.8072  Sham-CS veh vs Sham-CS THC: X^2^_(2)_ =4.258, p=0.1189  *Stress-NS veh vs Stress-NS THC: X^2^_(2)_ =12.43, p=0.002  *Stress-CS veh vs Stress-CS THC: X^2^_(2)_ =9.524, p=0.002 | |
| C |  |  | Veh  Pearson r=-0.05649, p=0.4345 | THC+CBD  *Pearson r=0.6499, p=0.0111 |
| D |  |  | Veh  Spearman r=0.0911, p=0.3952 | THC+CBD  *Spearman r=-0.5827, p=0.025 |
| E |  |  | **LMM (left)**  Number of Value: 175  Random effects  σ^2^animal=0.033; σ^2^residual=0.1615; ICC= 0.17  Fixed effects: Treatment group  *X^2^_(5)_= 11.9, p=0.0361, R^2^_m_=0.1288 | |
| F |  |  | **Multiple Chi-square; Corrected p =0.0071**  *Sham-CS veh vs Stress-CS veh: X^2^_(2)_ =10.19, p=0.0061  *Sham-CS veh vs Stress-NS veh: X^2^_(2)_ =28.2, p<0.0001  *Sham-CS THC vs Stress-CS THC: X^2^_(2)_ =53.07, p<0.0001  *Sham-CS THC vs Stress-NS THC: X^2^_(2)_ =18.58, p<0.0001  *Sham-CS veh vs Sham-CS THC: X^2^_(2)_ =19.02, p<0.0001  *Stress-NS veh vs Stress-NS THC: X^2^_(2)_ =22.66, p<0.0001  *Stress-CS veh vs Stress-CS THC: X^2^_(2)_ =46.39, p<0.0001 | |
| **Figure S4: Acute stress induced a constitutive astrocyte atrophy, which is reversed by stress-CS exposure only in female vehicle rats** | | | | |
|  | Total n | N break down | Statistical analysis | |
| A | 23 | Veh  Sham-CS =4; Stress-NS =4; Stress-CS =3  THC+CBD  Sham-CS =4; Stress-NS =5; Stress-CS =3 | **LMM (left)**  Number of Value: 175  Random effects  σ^2^animal=0.024; σ^2^residual=0.1318; ICC= 0.15  Fixed effects: Treatment group  *X^2^_(5)_= 17.7374, p=0.0033, R^2^_m_=0.1695 | |
| B |  |  | **Multiple Chi-square; Corrected p =0.0071**  Sham-CS veh vs Stress-CS veh: X^2^_(2)_ =0.4706, p=0.7903  *Sham-CS veh vs Stress-NS veh: X^2^_(2)_ =0.32.85, p<0.0001  *Sham-CS THC vs Stress-CS THC: X^2^_(2)_ =21.13, p<0.0001  *Sham-CS THC vs Stress-NS THC: X^2^_(2)_ =27.65, p<0.0001  Sham-CS veh vs Sham-CS THC: X^2^_(2)_ =14.33, p=0.2419  Stress-NS veh vs Stress-NS THC: X^2^_(2)_ =6.185, p=0.0454  *Stress-CS veh vs Stress-CS THC: X^2^_(2)_ =13.78, p=0.001 | |
| C |  |  | **LMM (left)**  Number of Value: 175  Random effects  σ^2^animal=0.04; σ^2^residual=0.1996; ICC= 0.17  Fixed effects: Treatment group  *X^2^_(5)_= 32.2458, p<0.0001, R^2^_m_=0.2802 | |
| D |  |  | **Multiple Chi-square; Corrected p =0.0071**  Sham-CS veh vs Stress-CS veh: X^2^_(2)_ =6.417, p=0.0404  *Sham-CS veh vs Stress-NS veh: X^2^_(2)_ =53.19, p<0.0001  *Sham-CS THC vs Stress-CS THC: X^2^_(2)_ =29.12, p<0.0001  *Sham-CS THC vs Stress-NS THC: X^2^_(2)_ =62.02, p<0.0001  Sham-CS veh vs Sham-CS THC: X^2^_(2)_ =7.116, p=0.0285  Stress-NS veh vs Stress-NS THC: X^2^_(2)_ =2.726, p=0.2560  Stress-CS veh vs Stress-CS THC: X^2^_(2)_ =4.758, p=0.0926 | |
| **Figure S5: MMP-2,9 activity regulated the astroglial plasticity following THC+CBD use in females** | | | | |
|  | Total n | N break down | Statistical analysis | |
| A | 29 | Veh  Sham-CS =4; Stress-NS =4; Stress-CS =5  THC+CBD  Sham-CS =5; Stress-NS =5; Stress CS =6 | Two-way ANOVA  Drug: F_1,23_=1.071, p=0.3115 Stress: F_2,23_=0.7095, p=0.5024  Drug x Stress: F_2,23_=1.002, p=0.3825 | |
| B | 18 | Veh  Sham-CS =2; Stress-NS =3; Stress-CS =2  THC+CBD  Sham-CS =3; Stress-NS =5; Stress-CS =3 | Veh  Pearson r=-0.1992, p=0.3343 | THC+CBD  *Pearson r=0.5972, p=0.0202 |
| C |  |  | Veh  Pearson r=-0.1375, p=0.3844 | THC+CBD  *Pearson r=-0.6611, p=0.0096 |

**Table S2: Full statistics for each figure.**
