# Supplementary material for: Cannabinoid use generalizes stress responses: involvement of astrocyte plasticity and activation of matrix metalloproteinases in the nucleus accumbens core
Source: Mol Psychiatry. 2026 Jun 11;31(10):5979–92. doi: 10.1038/s41380-026-03687-0 (PMC13569448; doi:10.1038/s41380-026-03687-0)
Supplement: Supplementary file 3 [file 41380_2026_3687_MOESM3_ESM.docx]

**Cannabinoid use generalizes stress responses: Involvement of astrocyte plasticity and activation of matrix metalloproteinases in the nucleus accumbens core**

**Supplementary methods**

**Acute Restraint Stress and Odor Pairing**

A total of 98 male and female Long-Evans rats were used to ensure that each treatment group had at least 4 subjects rats. Rats were randomly assigned to either the sham or stress group. At approximately 8 weeks of age (± 1 week; young adult), rats from the stress group were placed in flat-bottom restrainers (PLAS labs, Thomas Scientific, Swedensboro, NJ, USA), which limit movement but allow normal breathing. Then, rats were placed in another cage for 2 hours with a Petri dish filled with approximately 1 ml of an essential oil (lemon or sandalwood; Sun Essential, Phoenix, AZ, USA). This odor became the Stress-CS. In another room, control rats were exposed to the same odor for 2 hours in a new cage (Sham-CS).

**Drugs and reagents**

Both (-)-trans-Delta9-tetrahydrocannabinol (THC) (5mg/ml) in ethyl alcohol (95%) and Cannabidiol powder (CBD) (ADL-19496-11) are supplied by National Institute of Drug Abuse (NIDA) Drug Supply Program. The CBD was dissolved in pure ethanol at 20 mg/ml and was added to THC in a 10:1 ratio. The Intravenous self-administration solution was prepared by mixing THC+CBD with Tween-80 and sterile saline in a 1:1:83 ratio. The vehicle was made identically without THC+CBD. Dye-quenched FITC-gelatin (Thermo Fisher; D12054) was dissolved in 1X phosphate-buffered saline at 1mg/ml. MMP-2 (444288, MilliporeSigma) and MMP-9 (444278, MilliporeSigma) inhibitors were dissolved in 1% DMSO.

**Defensive Burying Task**

A standard home cage (18.5 x 10 x 8.5) was divided into two equal sections. Bedding was placed in one half, while the dish containing the odor was set in the other half. Before each DBT session, the dish was filled with about 1 ml of the essential oil. The odor previously paired with the stress/sham procedure represent the CS (Sham-CS or Stress-CS group) while the unpaired odor represents the neutral stimulus (Stress-NS group). To prevent the blending of different odors during the DBT, some rats were stressed and sham-exposed with lemon odor, while others were stressed with sandalwood odor. Consequently, on the test day, only the lemon odor was used as the CS and NS. Each rat was placed on the bedding side, facing the odor, and behavior was digitally recorded for 15 minutes. The immobility parameter was automatically scored by Ethovision XT while burying, grooming and escape were manually scored in a blinded manner using the Ethovision manual scoring tool. The experiment was replicated across 6 different cohorts of rats.

**In vivo zymography**

In vivo zymography is a technique used to quantify the gelatinolytic activity of MMP-2,9 in awake behaving rats. To this end, a volume of 1.5 µl of dye-quenched FITC-gelatin was injected into the NAcore right hemisphere through guide cannulae at 0.5 µl/min, followed by a 3-min diffusion period. The microinjectors used were 2mm longer than the guide cannulae to reach the targets. Rats started the DBT immediately after the diffusion period. A separate cohort of rats received bilateral infusion into NAcore of either DMSO, MMP-2 or MMP-9 inhibitors (0.5 µl, 0.25µl/min, 2min diffusion), 10 min before the dye-quenched FITC-gelatin microinjection. Immediately after the DBT, rats were perfused with 4% PFA, and brains were sliced at 100µm. Right hemisphere NAcore sections were mounted and coverslipped, then imaged using a Leica SP5 confocal microscope (see below).

**Confocal microscopy imaging**

MMP activity images were acquired with the 10X objective lens at 12-bit resolution with a 1-μm step size. The first and last optical sections of the Z-stack represented the beginning and the end of the fluorescence signal, respectively. Only slices displaying both the injection track and the anterior commissure on the same frame were imaged and analyzed (ImageJ; National Institute of Mental Health, Bethesda, MD). The tissue slice with the highest integrated density was selected for each animal. The fluorescence intensity directly indicates the gelatinolytic activity of MMP-2,9.

Astrocytes were acquired with a 63X oil-immersion objective lens with the following parameters: 16-bit resolution, 1-μm step size, 1024 x 1024 frame size, and 4-frame averaging. Between 4 and 11 astrocytes were imaged per rat, using 2 to 4 slices for each rat. Astrocytes were included only if the entire cell was visible within the section and its borders were clearly separate from adjacent labeled cells.

**Imaris 3D reconstruction**

Astrocyte images were imported into Imaris software (version 9.7.0), and the hM3dq-mCherry signal from each astrocyte was used to generate a 3-dimensional reconstruction of astrocytes using the Surface module. This 3D reconstruction was used to generate a new channel that isolates the mCherry astrocyte from the background, which was then designated as a region of interest (ROI). The Synapsin-I channel was intensity-thresholded to isolate Synapsin-I puncta from noise. The Colocalization module was used to determine the percentage of the ROI that colocalized with Synapsin-I puncta. The Synapsin-I density was measured by normalizing the number of Synapsin-I puncta to the volume of the frame from which it was obtained.

**Statistics**

All data were analyzed using GraphPad Prism 10 and Python with the statsmodels library. One outlier identified with ROUT (Q=1%) was removed from the study. Two-way repeated-measures ANOVAs were conducted to analyze self-administration data, with stress and days as independent variables and active lever presses, infusions, and the discrimination index as dependent variables. Two-way ANOVAs with Tukey’s post hoc tests were used to analyze behavioral and in vivo zymography data, with stress and drugs as independent variables and coping strategies as dependent variables. The D’Agostino-Pearson normality test was used to assess whether astroglial and Synapsin-I data were normally distributed. A linear mixed model (LMM) was used with non-normally distributed data to evaluate Fixed and Random effects. The Fixed effect was employed to analyze the influence of stress and THC+CBD use on the astrocyte-Synapsin-I association, Synapsin-I density, and astroglial morphology. Sham-CS vehicle-trained rats served as controls for each treatment group. The Joint Wald test was used to evaluate the main effect of the Fixed effects and the Wald chi-square post hoc test was used to compare the following groups: Sham-CS THC+CBD versus Stress-NS THC+CBD; Sham-CS THC+CBD versus Stress-CS THC+CBD; Stress-NS Veh versus Stress-NS THC+CBD, and Stress-CS Veh versus Stress-CS THC+CBD. Random Effects were used to estimate the within- (σ^2^residual) and between-animal (σ^2^animal) variances. An intraclass correlation coefficient (ICC) was calculated to estimate the proportion of variance attributable to differences between animals. Effect size was reported as eta-square (ƞ^2^) for two-way ANOVA analysis and marginal R-square (R^2^_m_) for LMM analysis. Separate Chi-square tests were performed for each behavior category, using Bonferroni correction to adjust for multiple comparisons. Pairwise comparisons include: Sham-CS Veh versus Stress-NS Veh; Sham-CS Veh versus Stress-CS Veh; Sham-CS THC+CBD versus Stress-NS THC+CBD; Sham-CS THC+CBD versus Stress-CS THC+CBD; Sham-CS Veh versus Sham-CS THC+CBD; Stress-NS Veh versus Stress-NS THC+CBD, and Stress-CS Veh versus Stress-CS THC+CBD.

**Code availability**

Python codes are available upon request from the corresponding author

**Supplemental figure legends**

**Figure S1: Acute restraint stress increased the discrimination index in female THC+CBD rats. A)** Active lever presses during THC+CBD self-administration in females. **B)** Daily intake of THC+CBD. **C)** The sham group showed no distinction between the active (THC+CBD infusion) and inactive (no consequence) levers during the final 5 days of self-administration (stress: F_1,15_=13.53, p=0.0022, ƞ^2^=0.26). The dotted line represents a 2:1 ratio of active to inactive. **D)** Acute stress did not affect the active lever presses during vehicle self-administration in females. **E)** Sham and stress groups consumed the same amount of vehicle. **F)** No difference was observed between sham and stress rats on the discrimination index during vehicle self-administration. See table S2 for complete analysis. Data are shown as mean ± SEM. Number of animals is expressed in brackets. *p< 0.05, comparing stress to sham.

**Figure S2: Coping strategies in females.** Stress and THC+CBD had no effect on **A)** burying, **B)** escape**, C)** immobility**, D)** grooming behaviors. Number of rats is expressed in **C.** See table S2 for complete analysis. Data are shown as mean ± SEM.

**Figure S3: THC+CBD use potentiated the astrocyte-synaptic association after the stress-CS exposure in females. A)** Stress-CS exposure increased astrocyte-Synapsin-I colocalization in both vehicle- and THC+CBD-trained rats (). Data are shown as mean ± SEM. N shown in legend as astrocytes/animals. * p< 0.05 compared to Sham-CS within each drug group; # p< 0.05 comparing THC+CBD to vehicle within each stress/sham group. **B)** The combination of prior acute stress and cannabinoid abstinence increased the subpopulation of astrocytes close to Synapsin-I (X^2^_(2)_ =12.43, p=0.002 versus Stress-NS veh), while stress-CS exposure almost completely eliminated astrocytes at mid-distance (X^2^_(1)_ =9.52, p=0.002 versus Stress-CS veh). See table S2 for complete analysis. * p< 0.05 compared to Sham-CS within each drug group; # p< 0.05 comparing THC+CBD to vehicle within each stress/sham group using a Bonferroni correction. **C)** Positive correlation between astrocyte-Synapsin-I colocalization and time spent burying in THC+CBD-trained rats (Pearson r=0.65, *p=0.011), not in vehicle-trained rats. **D)**  Negative correlation between the astrocyte-Synapsin-I colocalization and grooming in THC+CBD-trained rats (Spearman r=-0.582, *p=0.0252), not in vehicle-trained rats. **E)** THC+CBD abstinence increased Synapsin-I density in females with previous stress experience (X^2^_(5)_= 11.9, p=0.0361, R^2^_m_=0.1288). Data are shown as mean ± SEM. N shown in legend as astrocytes/animals. * p< 0.05 compared to Sham-CS within each drug group; # p< 0.05 comparing THC+CBD to vehicle within each stress/sham group. **F)** Astrocytes surrounded by lower Synapsin-I density represented a larger population in vehicle-stressed rats compared to Sham-CS (X^2^_(2)_ =28.20, p<0.0001 versus Stress-NS Veh; X^2^_(2)_ =10.19, p=0.0061 versus Stress-CS Veh). Abstinence from THC+CBD constitutively increased the astrocyte subpopulation surrounded by lower Synapsin-I density (X^2^_(2)_ =19.02, p<0.0001 versus Sham-CS Veh). See table S2 for complete analysis. * p< 0.05 compared to Sham-CS within each drug group; # p< 0.05 comparing THC+CBD to vehicle within each stress/sham group using a Bonferroni correction.

**Figure S4: Acute stress induced a constitutive astrocyte atrophy, which is reversed by stress-CS exposure only in female vehicle rats. A)** Acute stress decreased the surface area of astrocytes in both vehicle and THC+CBD-trained rats, which is restored by stress-CS exposure (X^2^_(5)_= 17.73, p=0.003, R^2^_m_=0.1695). Data are shown as mean ± SEM. N shown in legend as astrocytes/animals. * p< 0.05 compared to Sham-CS within each drug group; # p< 0.05 comparing THC+CBD to vehicle within each stress/sham group. **B)** THC+CBD prevented the restoration of the astrocyte distribution induced by stress-CS exposure (X^2^_(2)_ =21.13, p<0.0001 versus Sham-CS THC+CBD; X^2^_(2)_ =13.78, p=0.001 versus Stress-CS Veh) See table S2 for complete analysis. * p< 0.05 compared to Sham-CS within each drug group; # p< 0.05 comparing THC+CBD to vehicle within each stress/sham group using a Bonferroni correction. **C)** A prior traumatic event decreased the volume of astrocytes in both vehicle and THC+CBD-trained rats, while THC+CBD prevented the restoration of astroglia volume following stress-CS exposure (X^2^_(5)_= 32.25, p<0.0001, R^2^_m_=0.2802). Data are shown as mean ± SEM. N shown in legend as astrocytes/animals. * p< 0.05 compared to Sham-CS within each drug group; # p< 0.05 comparing THC+CBD to vehicle within each stress/sham group. **D)** Astrocyte subpopulation across all conditions. See table S2 for complete analysis. * p< 0.05 compared to Sham-CS within each drug group; # p< 0.05 comparing THC+CBD to vehicle within each stress/sham group using a Bonferroni correction.

**Figure S5: MMP-2,9 activity regulated the astroglial plasticity following THC+CBD use in females. A)** MMP-2,9 activity is not affected by stress or THC+CBD use. See table S2 for complete analysis. Data are shown as mean ± SEM. **B)** MMP-2,9 activity positively correlated with astrocyte-Synapsin-I colocalization after THC+CBD use (Pearson r=0.597, *p=0.02), not vehicle. **C)** MMP-2,9 activity negatively correlated with astroglial volume after THC+CBD use (Pearson r=-0.661, *p=0.009), not vehicle.

**Figure S6: Effect of MMP-2 and MMP-9 inhibitors on coping strategies. A)** Representative 63X confocal images of NAcore astrocytes. (1) Merged image of a GFAP-hM3dq-mCherry astrocyte (red) with the presynaptic marker Synapsin-I (blue). Scale bar=7µm. (2) Immunolabeled Synapsin-I channel used for the quantification of Synapsin-I density. (3) Digital rendering of the astrocyte in panel 1. (4) Digitally isolated Synapsin-I puncta that colocalized with the astroglial volume (white). **B)** MMP-9 inhibitor decreased the burying frequency (Kruskal-Wallis=7.69, p=0.0152). **C)** MMP inhibitors did not significantly affect the escape behavior (Kruskal-Wallis=3.873, p=0.1293). **D)** MMP inhibitors had no effect on immobility (Kruskal-Wallis=1.065, p=0.6013). **E)** MMP-2 inhibitor decreased the time spent grooming (Kruskal-Wallis=8.67, p=0.0076). Data are shown as mean ± SEM. Each dot in bar represents an analyzed astrocyte. * p< 0.05 compared to DMSO within each drug group using a Dunn post hoc; # p< 0.05 comparing 9i to 2i.
